# The emergence of the multisensory brain: From the womb to the first steps

**DOI:** 10.1016/j.isci.2023.108758

**Published:** 2023-12-15

**Authors:** Elena Nava, Michelle Giraud, Nadia Bolognini

**Affiliations:** 1 Department of Psychology & Milan Centre for Neuroscience (NeuroMI), University of Milan-Bicocca, Milan, Italy; 2 Laboratory of Neuropsychology, IRCCS Istituto Auxologico Italiano, Milan, Italy

**Keywords:** Behavioral neuroscience, Developmental neuroscience, Sensory neuroscience

## Abstract

The becoming of the human being is a multisensory process that starts in the womb. By integrating spontaneous neuronal activity with inputs from the external world, the developing brain learns to make sense of itself through multiple sensory experiences. Over the past ten years, advances in neuroimaging and electrophysiological techniques have allowed the exploration of the neural correlates of multisensory processing in the newborn and infant brain, thus adding an important piece of information to behavioral evidence of early sensitivity to multisensory events. Here, we review recent behavioral and neuroimaging findings to document the origins and early development of multisensory processing, particularly showing that the human brain appears naturally tuned to multisensory events at birth, which requires multisensory experience to fully mature. We conclude the review by highlighting the potential uses and benefits of multisensory interventions in promoting healthy development by discussing emerging studies in preterm infants.

## Graphical abstract

**Figure:**
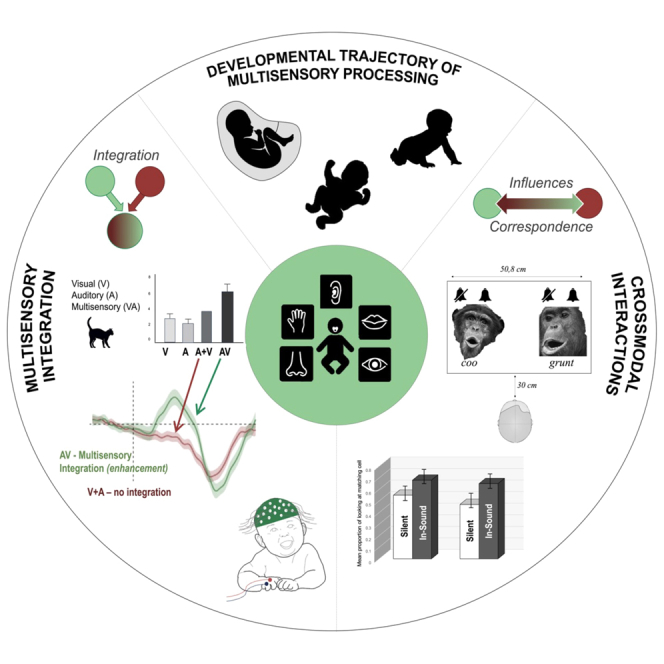

## Early sensitivity to multisensory events: Insights from behavioral newborn studies

Growing evidence shows that a large part of the human brain is intrinsically multisensory, that is, able to process and integrate sensory information regardless of its modality, not only in higher order association cortices but even at a very low-level of processing, in the putatively modality-specific primary sensory areas.^1^^,^^2^ The multisensory organization of the cerebral cortex has been supported by neurophysiological studies in animals, showing the existence of multisensory neurons in various brain structures, as well as direct feedforward connections between the primary sensory cortices and feedback pathways from higher-order cortical regions and low-level sensory areas and subcortical structures.^3^^,^^4^^,^^5^

At the behavioral level, the primary advantage of multisensory integration consists in the enhancement of the salience of sensory stimuli which, in turn, facilitates behavioral responses to them.^6^ The term ‘multisensory integration’ is typically used to refer to situations in which multiple stimuli presented in different sensory modalities, but in close spatial and temporal proximity, are bound together,^7^^,^^8^ mimicking the multisensory enhancement effects observable at the cellular level; instead, stimuli from different sensory modality are not integrated into a unified percept, and may even hamper sensory processing, if spatially and temporally incongruent.

Indeed, of particular relevance to understanding how our brain faces incoming multiple sensory information has been the discovery of multisensory neurons in the cat’s superior colliculus (SC), so much that it became a representative model to study multisensory integration in the human brain and perceptual systems.^3^ Most importantly, these studies have served the developmental cause, documenting that, first, the multisensory layers of the SC of newborn animal are not silent at birth but become overtly responsive to sensory stimuli depending on the time in which the single organs become ‘active’. In other words, at birth the cat’s SC neurons are only responsive to tactile stimulation because it is the only afference at that stage of development; the ear canals and eyelids open approximately 10 days after birth, and around this time, auditory and visual responsiveness appear too. However, the ability to synthesize such different sensory information (indexed by the enhancement of neural activity by multisensory stimuli) appears weeks later, suggesting that the animal requires a more prolonged sensory experience to optimize multisensory processing through a shared neural code.

This view is supported by neurophysiological evidence in experimentally deprived animals (e.g.,^9^): cats reared in the dark up to 6 months of age present a certain degree of immaturity of multisensory neurons, which indeed not benefit from multisensory stimulation, so that their responses to congruent audiovisual stimulation is not greater to their response to unisensory stimuli (auditory or visual), which corresponds to impairments of behavioral responses to combination of sensory stimuli (see^10^).

This evidence converges with that in cataract-reversal patients, who represent the corresponding human model to the cats reared in the dark. Indeed, these individuals are born blind due to cataract, but their vision can be restored through surgery following even a short period of visual deprivation. However, restoring vision even within the first year of life has long-term consequences on the development of multisensory capabilities, in that these patients present impaired multisensory processing in adulthood, at net of their visual performance, which develops typically. These findings, together with the animal studies mentioned above, suggest that multisensory input is necessary during the first weeks of life for the full maturation of multisensory interactions (see^11^^,^^12^).

To date, studies in newborns investigating multisensory integration as defined above are sparse, while there is evidence that, from the very first hours of life, newborns are sensitive to crossmodal interactions, both for low-level object characteristics (e.g., shape, see^13^) and for social stimuli, such as the matching between the mother’s voice and face.^14^ The term crossmodal interaction is not synonymous to multisensory integration, since it refers to situation in which stimuli from a sensory system can exert an influence on the perception of, or the ability to respond to, stimuli presented in another sensory modality, without resulting in a unified representation directly traceable to the activity of multisensory neurons.^8^ This process reflects the dynamic exchange of information between sensory systems, without necessarily involving the convergence of different sensory inputs on a single neuron. At the behavioral level, crossmodal interactions may cause perceptual distortions or illusory effect, occurring when a discordant sensory input overpowers another one that is less reliable,^7^ or they may favor the matching of distinct features or dimensions of experience across different senses (i.e., crossmodal correspondence).

The study of crossmodal interactions in newborns has capitalized on classic preferential looking techniques, by which longer looking/visual attention or the number of head turns directed toward one stimulus over another is taken as a marker that newborns not only can discriminate between two stimuli but actually prefer one over the other. Worth noting, a preference for paired bimodal stimuli over unimodal stimuli can also be seen as evidence of multisensory facilitation in processing, for example, the mother’s face (as seen in^14^).

**Figure 1:**
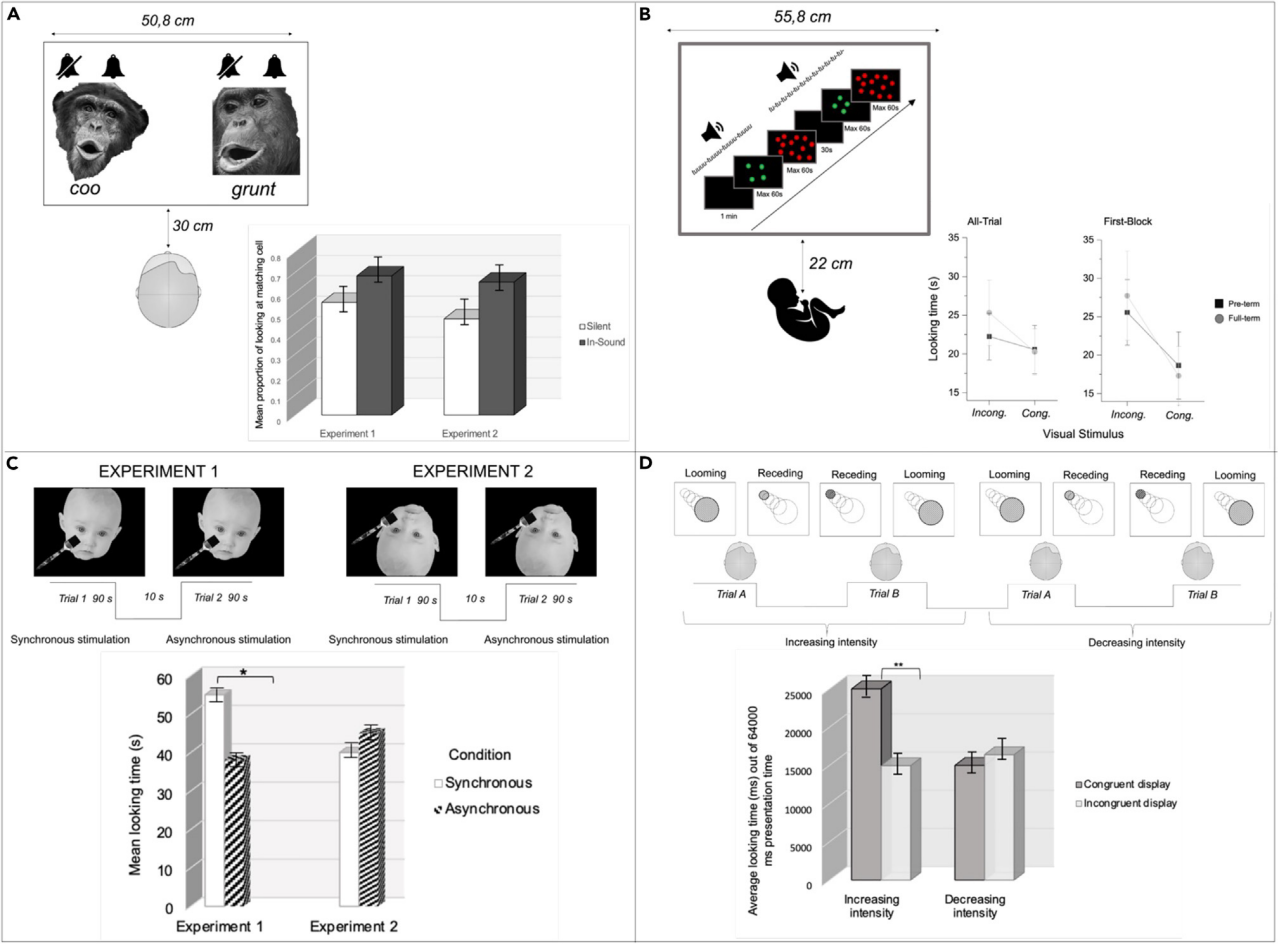
Graphical adaptation of the results of behavioral studies depicting examples of multisensory interactions at birth and early infancy (A) Displays the study by Lewkowicz et al.^15^ (2010), showing that multisensory perceptual tuning is broad at birth, enabling them to integrate facial and vocal gestures of non-native social stimuli. (B) Displays the study by Anobile et al.^17^ (2021), revealing that multisensory cues help them matching audiovisual stimuli related to numbers. (C) Displays the study of Filippetti et al.^18^ (2013), in which newborns showed a preference for infant’s faces while being stroked on their cheek, but only when the image was presented upright. (D) Displays the study of Orioli et al.^19^ (2018), showing that newborns can match auditory and visual stimuli that apparently move toward or away from them.

Interestingly, work from Lewkowicz et al.^15^(2010, see Figure 1, panel A) has shown that crossmodal interactions are broadly tuned in early infancy; this translates, for example, into newborns’ ability to match not only native faces and voices but also non-native social stimuli, such as a non-human primate facial and its vocal gestures. Indeed, newborns look longer at a non-human primate face, producing a visible call that is accompanied by a corresponding audible call, with respect to a face not producing any sound (Experiment 1) and a face that produces a complex tone different from the corresponding one (Experiment 2). Note that this broad crossmodal interaction disappears with age due to ‘perceptual narrowing’, a concept that reflects the growing specialization of the brain in its native environment.^16^ Thus, by the end of the first year of life, infants lose their ability to discriminate between non-native stimuli in favor of increasing capabilities of discriminating across native stimuli (e.g., identities, phonemes, etc).

Another line of investigation has focused on the role of crossmodal information in the development of abstract concepts, such as number.^17^^,^^20^ By adapting a behavioral paradigm developed by Izard et al.^20^ (2009), Anobile et al.^17^ (2021, see Figure 1, panel B) have explored crossmodal influences on number cognition. Auditory sequences containing a fixed number of sounds (i.e., 4 or 12 syllables) were presented to newborns: In the first minute of the auditory stimulation, the screen remained black; then, two consecutive images containing the same or a different number of items were displayed. The results revealed that newborns looked longer at the incongruent visual numerical stimuli, suggesting that audiovisual interactions favor the development of numerical cognition.

Crossmodal interactions were also explored with respect to the development of body representation; in human adults, body representation relies on the stability of multisensory cues for the construction and maintenance of the self.^21^ For example, Filippetti et al.^18^ (2013, see Figure 1, panel C) presented newborns with videos of either upright or inverted infant faces, who were stroked with a paintbrush on one of their cheeks. The newborn participants were stroked on one cheek by the experimenter, either synchronously or asynchronously, with respect to the viewed infant’s face. The results showed that newborns preferred the synchronous condition, but only when the viewed face was upright. That is, newborns do not prefer the amodal properties of multisensory stimuli *per se* (i.e., synchrony of observed and felt stroking) but prefer the congruence with their own body, i.e., prefer a body that looks like their own or is, at least, in an anatomically possible position (upright).

Another study by Filippetti et al.^22^ (2015) showed that newborns prefer synchronous visuo-tactile stimulation only if the seen and felt touch is spatially congruent, as shown in adults (e.g.,^23^^,^^24^). Newborns were presented with videos of infant faces touched either on their cheeks or foreheads. Newborns received the touches always synchronously to the viewed touch, they could be touched on the same/congruent body part (e.g., cheek-cheek) or on a different/incongruent body part (e.g., cheek-forehead) with respect to the viewed face. Newborns preferred the congruent over the incongruent visuo-tactile stimulation, and these data, together with the previous studies by the same authors, corroborate the notion that soon after birth, newborns are equipped with a rudimentary sense of bodily self, the origins and mechanisms of which are rooted in the temporal coherence of multisensory stimuli.

Again, related to body representation and perception, a recent study showed that newborns prefer approaching stimuli and do not show any preference for receding stimuli (^19^, see Figure 1, panel D). This was investigated by presenting newborns with two side-by-side visual displays, one looming toward and the other receding from the participant. A sound that either increased or decreased in intensity accompanied the visual looming stimulus, thus creating a congruent audiovisual approaching (i.e., a visual stimulus increasing in size accompanied by a sound rising in intensity) or receding (i.e., a visual stimulus decreasing in size accompanied by a sound decreasing in intensity) stimulus. Newborns only looked longer at the visual display when the sound increased in intensity but not when it decreased, thus revealing a preference for approaching audiovisual stimuli. This pattern is interesting with respect of two aspects of crossmodal correspondence: first, newborns are able to match dynamic, congruent, audiovisual information in which no redundant amodal feature is provided; second, since this crossmodal matching occurred only for approaching stimuli suggests that since the early stages of life, newborns are equipped with some multisensory knowledge about their own body, and can ‘recognize’ the evolutionary meaning of a looming stimulus approaching the body, often fundamental to activate defensive reactions.

While these studies have all provided evidence in favor of the notion that newborns appear to be tuned to multisensory events from very early on in ontogeny, only a few studies have investigated the neural correlates of such precocious ability. Because of the scarcity of studies conducted in newborns, the following paragraphs will discuss studies conducted in the first year of life and the contribution of different methods in unveiling the development of the multisensory human brain.

We conclude the review with a paragraph on the role of multisensory interventions in promoting well-being in preterm infants, highlighting the benefit of multisensory processing early in life and the importance of investigating its functioning from a behavioral and a neurophysiological point of view.

The idea of multisensory intervention is familiar and has been explored in both typical and atypical development. In particular, for the former, there is evidence that multisensory training can translate in better performance even in unisensory tasks.^25^^,^^26^ For example, individuals trained with audiovisual stimuli show improvement of both visual and auditory processing alone. This phenomenon also extends to social stimuli, as is the case of individuals trained with faces and voices, who are then facilitated in recognizing a voice without any visual cue,^27^ or individuals trained to encode objects with multisensory features, who then more easily process the same objects even under unisensory conditions.^28^

Of particular interest is the suggestion that even a mere multisensory experience - without any training – may impact on higher-level cognitive functions, such as memory and reasoning. Denervaud et al.^29^ (2020) provided evidence for this in a small group of children and adolescents, who underwent a simple detection task with unisensory (visual or auditory stimuli presented alone) and multisensory (audiovisual) stimuli and a multisensory recognition task, the discrimination of common objects presented with or without auditory congruent stimuli. These two measures of multisensory abilities were then correlated with the children’s working memory and abstract non-verbal reasoning performance. The first was measured with a backward digit span, the second by means of the Raven’s Colored Progressive Matrices. The results showed that multisensory abilities (in term of multisensory integration and crossmodal interactions effects) predict memory and intelligence scores. Although these data do not demonstrate the causal role of multisensory experience in promoting cognitive functioning, they at least suggest that multisensory processes are associate to mental processes. These findings are further supported by evidence in typical aging population: older people with better multisensory integration abilities present higher cognitive profiles on tasks of sustained attention, memory, processing speed, and executive functions.^30^

The fact that multisensory abilities are tightly linked to cognitive processes suggests that potentiating multisensory mechanisms might also improve cognitive processes; this, in turn, opens a new avenue of research, particularly for studying the effects of such training in preterm infants, who have an increased risk for learning disabilities but whose future outcome might be protected by early interventions.^31^^,^^32^^,^^33^

## Electroencephalographic studies (EEG)

### Audiovisual interactions

Among the many multisensory experiences, seeing talking faces is likely the most immediate and intense stimulation infants experience since birth. To observe the neural signatures of audiovisual speech perception, Hyde et al.^34^ (2011) presented 5-month-old infants with a female face saying ‘hi’; the auditory component, presented 400 ms before the face onset, could be synchronous or asynchronous with respect to the face.

Interestingly, but contrary to what is commonly observed in adults in which there is a modulation of EEG activity by synchronous stimuli (e.g.,^35^^,^^36^), infants exhibited greater magnitude of a large slow negative component (Nc) over frontocentral sites in the asynchronous, but not the synchronous, condition, suggesting that infants find this event particularly unfamiliar and novel and thus captures their attention.

Importantly, the ‘perceptual narrowing’^16^^,^^37^ mentioned above proposes that the developmental process ‘pushes’ toward the specialization of the perceptual systems and, by doing so, progressively sharpens the perceptual abilities of the individual. From a behavioral and functional point of view, this translates into ‘being able to do less, but better’ within a certain developmental period and likely corresponds to the pruning of unused connections in favor of the strengthening of connections that are highly used. For example, Pons et al.^38^ (2009) showed that 6-month-old native Spanish infants can match audiovisual speech with English speech sounds but lose this ability by the age of 11 months, as they have developed a specialized system for detecting and discriminating their native language.

The neural correlates of such narrowing were investigated by Grossmann et al.^39^ (2012), who recorded event-related potentials (ERPs) in 4- and 8-month-old infants while they viewed a human and a non-human primate face, producing a facial expression that could either match or non-match the heard vocalizations ‘grunt’ and ‘coo’. The electrophysiological responses mimicked the ones observed in the behavioral studies, namely 4-month-old infants were sensitive to the congruent match (face and voice), irrespective of face species (human and non-human primate); on the contrary, 8-month-olds were only sensitive to the congruent human face and vocalization but did not detect the incongruency between non-human primate face and vocalization. In particular, ERP analyses revealed recruitment of the N290 (a typical component signaling face processing in early infancy, see^40^) in both younger and older infants; however, while there was no difference between human and non-human primate faces in 4-month-olds, the N290 in 8-month-old infants was more negative for non-human primate than human faces, revealing the emergence of a specialized system for own-species faces that starts by 8 months of age. This study, and the general idea of perceptual narrowing suggest that at birth, humans are broadly tuned to multisensory information; however, postnatal experience with one’s own species is associated with neural changes in multisensory processing.

### Odor-visual

Category acquisition in the first months of life seems to be promoted by multisensory integration, with early maturing systems, such as olfaction, driving the acquisition of categories in later-developing systems, such as vision. In their elegant study, Rekow et al.^41^ (2021) provided original evidence to support that view. Fast periodic visual stimulation of natural images of objects, as well as face-like stimuli, was coupled with EEG frequency tagging to track visual responses to the category ‘face’ in infants. The presentation of the images was accompanied by two odors, one maternal and one neutral (control/baseline). Within the sequence, face-like images were interleaved at every sixth stimulus, corresponding to a periodic rate of 1 Hz. This period response in the EEG frequency spectrum commonly reflects a response that is specific to faces and not to the stimulus category displayed within the sequence. The results revealed that by 4 months of age, infants’ ability to categorize face-like stimuli are initiated by maternal odor. Indeed, cerebral activity is comparable between the two hemispheres when infants are presented with the control odor. However, when coupled with maternal odor, cerebral activity is larger over the right than the left hemisphere, where the specialized response to faces usually occurs in adults (e.g.,^42^). Thus, the developing visual system builds upon multisensory experiences (e.g., maternal odor and face-like stimuli) to learn to categorize complex unimodal sensory inputs, such as faces.

An important finding of Rekow et al.’s study^41^ relates to individual differences in reactions to multisensory events: the infants who presented the weakest responses in the baseline odor condition had the largest visual responses with the maternal odor. This effect is reminiscent of the inverse effectiveness principle, by which, at the neural level, maximal multisensory response enhancement occurs when the constituent unisensory stimuli are minimally effective in evoking responses.^43^^,^^44^ Applied to the current data, it would indicate that the more ambiguous a visual stimulus is to the infant, the more the maternal odor can promote face categorization, suggesting that multisensory input is crucial to developing specialized systems.

### Audio-tactile

**Figure 2:**
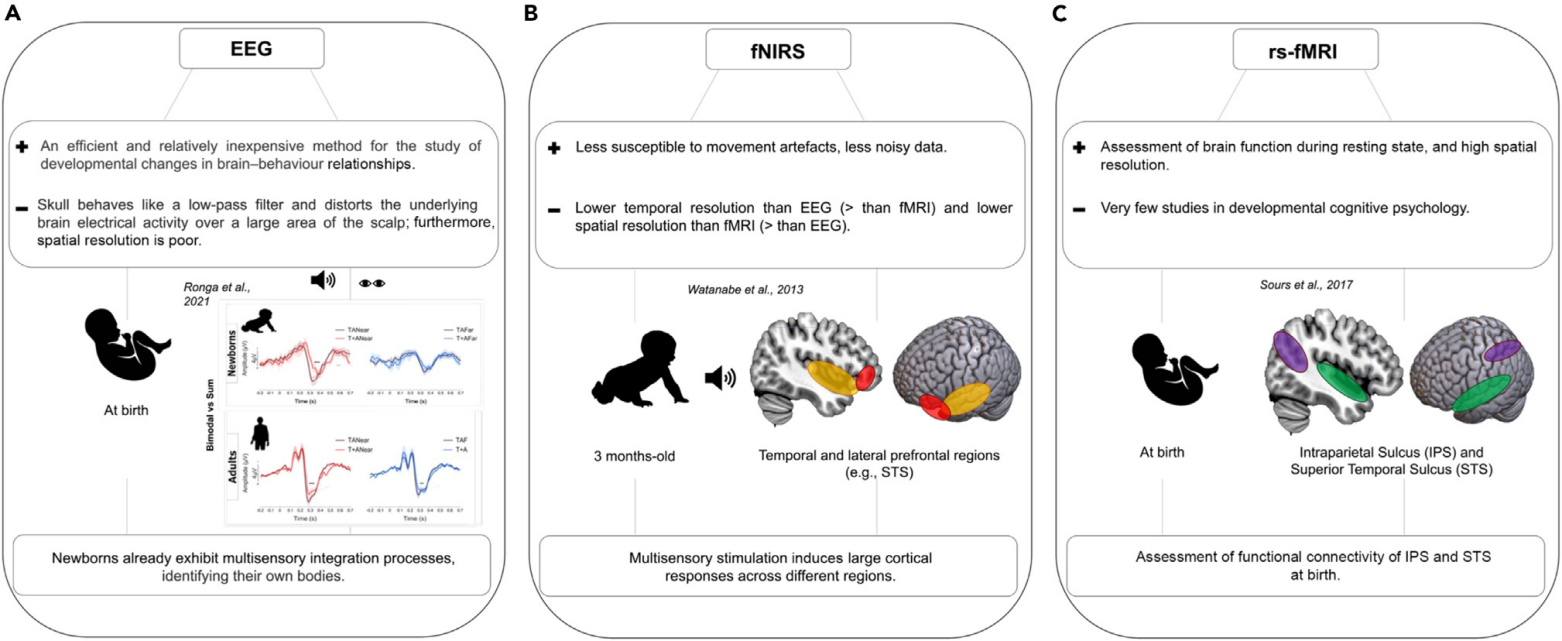
Comparison of three neurophysiological techniques used to assess multisensory interactions in newborns (A) Displays the study by Ronga et al.^45^ (2021), in which newborns’ electrophysiological response (EEG) to audio-tactile stimuli was recorded when presented close or far from the body. The authors found an electrophysiological pattern of MSI, with older newborns showing a larger MSI effect. (B) Shows the fNIRS study of Watanabe et al.^47^ (2013) aimed at assessing spatiotemporal cortical hemodynamic responses of 3-month-old infants while they perceived visual objects with or without accompanying sounds. The comparison between the two stimulus conditions revealed that the effect of sound manipulation was pervasive throughout the diverse cortical regions, and the effects were specific to each cortical region. Finally, (C) shows the rs-fMRI study by Sours et al.^48^ (2017), in which the functional connectivity of two typical multisensory cortical areas was found to be functional at birth: the intraparietal sulcus and the superior temporal sulcus.

Finally, it is worth mentioning a recent newborn study that investigated the neural correlates of the peripersonal space using EEG, which is closely linked to body representation. Ronga et al.^45^ (2021) touched one of the hands of the newborns while presenting auditory stimuli near or far from the touched hand (see Figure 2, panel A). Unisensory and multisensory stimuli were presented either close or far with respect to the body, under the assumption that stimuli occurring close to the body might speed up neural activity^46^ (i.e., peripersonal space has a protective function and events occurring within it are more relevant to the organism). In fact, newborns showed significantly multisensory integration effects for stimuli presented in near space, specifically in a time window between 222 ms and 338 ms post-stimulus onset, which corresponds to the latency of the P2 component. Overall, this study shows that newborns already exhibit multisensory integration processes active at birth, which can be detected not only from preferential looking times studies,^9^ but also at the level of their neural activity.

## Functional near-infrared spectroscopy (fNIRS)

Functional near-infrared spectroscopy (fNIRS) represents a relatively new neuroimaging technique well-suited to study infant brain responses to basic sensory stimuli, as well as to more complex interactive social contexts^49^ (see^50^ for a review of the technique). fNIRS measures cerebral functions through different chromophore mobilization (oxygenated hemoglobin, deoxygenated hemoglobin, and cytochrome c-oxidase, which absorb light at different wavelengths) and their timing with events/stimuli. With respect to its use in developmental neuroscientific research, the major advantage of fNIRS is that its data output is less susceptible to infants’ movement artifacts than other techniques. With regard to temporal resolution, it is higher than fMRI but lower than EEG; on a spatial resolution level, it is higher than EEG but lower than fMRI.

Despite these shortcomings, fNIRS has been increasingly used in developmental research, including recent studies investigating the multisensory nature of cortices in early infancy.

Watanabe et al.^47^ (2013) measured fNIRS responses in 3–4 months infants while they saw visual objects presented alone or with a concurrent sound (Figure 2, panel B). Under the assumption that increases in the oxy-Hb signal and decreases in the deoxy-Hb signal indicate cortical activation (positive), while a decrease in the oxy-Hb signal and increase in the deoxy-Hb signal indicate cortical deactivation (negative), the data showed positive changes in oxy-Hb signals in the audiovisual condition in the earliest time window in the temporal auditory regions and persisted until the later windows (up to 13 s following stimulus onset) in the early visual, posterior temporal, and prefrontal areas. Instead, in the unisensory condition, where only the visual stimulus was present, positive changes in oxy-Hb signals were observed at temporal and lateral prefrontal regions in early time windows but disappeared later.

In general, it should be noted that the presence or absence of sound dramatically affected the activation and/or deactivation of the diverse cortical regions. Furthermore, the difference between sound and no-sound conditions was compared within different brain regions, revealing different activation and deactivation patterns depending on the cortical site. For example, while the superior temporal sulcus (STS) and the middle temporal gyrus (MTGa) showed activation with large amplitudes in the sound condition and deactivation in the later time windows in the no-sound condition, the inferior frontal gyrus showed equal activation in both conditions.

The major finding that emerged from Watanabe et al.’ study^47^ (2013) was that in 3/4-month infants, multisensory stimulation induces large cortical responses across multiple regions, including heteromodal association areas, namely: the middle temporal gyrus (MTG), middle occipital gyrus (MOG), and inferior parietal lobe (IPL) as well as prefrontal regions inferior frontal gyrus (IFG) and superior frontal gyrus (SFG).

Importantly, the comparison between the addition vs. removal of auditory feedback to the visual image captured important characteristics of multisensory processing in infancy: auditory input did not just enhance overall activation concerning visual perception but also induced specific changes in each cortical region. For instance, removing the sound produced active suppression of activity in the superior temporal gyrus (STG, an early auditory region) and MTG. Removal of the sound also failed to produce any activity in the SFG, revealing that this region only responds to multisensory information. Similarly, removing the sound reduced the activation of MTG, MTG and IPL, revealing the multisensory properties of these regions and demonstrating that their functions are already in place very early in development.

FNIRS studies have also begun to shed light on brain connectivity, subtending multisensory processing in infancy. For example, Werchan et al.^51^ (2018) presented familiarization events in which infants viewed a ball bouncing either up and down or back and forth across the screen, while a sound was presented either as the ball changed direction in the synchronous condition, or 450 ms before the ball changed direction in the asynchronous condition. Before and after these familiarization events, infants saw two unisensory events: the bouncing ball without sound (unisensory visual) and the sound alone without the ball (unisensory auditory). One hypothesis of the authors was that if the occipital and temporal regions are functionally connected during multisensory synchronous events, this would suggest that crossmodal interactions might become an experience-driven process by which the unisensory cortices somehow ‘train’ each other to become multisensory. Accordingly, the results showed that infants with higher occipitotemporal connectivity exhibited occipital activation to unisensory auditory stimuli, thus revealing the multisensory properties of the occipital cortex. Although the temporal cortex did not exhibit the same pattern, the results nevertheless support the notion that functional connectivity enhances sensory processing by amplifying neural responses in unisensory cortices.^52^ Because the unisensory cortices did not respond to multisensory stimuli, this study suggests the possibility that multisensory processing, rather than being innate, represents a learned response to the naturally multisensory environment in which the child grows up.

## Localizing multisensory areas in the infant brain through resting-state Functional Magnetic Resonance imaging (rs-fMRI)

Despite its great potential and general interest, the use of resting-state Functional Magnetic Resonance (rs-fMRI) in developmental studies is still in its infancy. fMRI is a non-invasive brain functional imaging technique that can provide relatively high spatial resolution compared with the previous techniques mentioned (EEG and fNIRS). While fMRI provides BOLD signals of task- and cognitive-related stimuli, rs-fMRI assesses brain functions during the resting state, thus reflecting spontaneous neural activity. This activity is believed to relate to naturally existing brain organization, supported by vast cerebral connectivity,^53^ forming a coordinated BOLD fluctuation pattern (also known as ‘FC’). Spontaneous brain activity supports a series of behaviors, such as preparing for task execution^54^ or consolidating memories.^55^

The benefits of using rs-fMRI in the neonatal population appear almost obvious: task-free recording of brain activity allows establishing the existence of neural networks in a population that can only indirectly perform a task (e.g., assessment of visual preference). A recent rs-fMRI study^48^ explored the functional connectivity of typical areas of multisensory convergence in human adults - the intraparietal sulcus (IPS) and STS – at birth to prove the innateness of the cortical multisensory architecture. The IPS is involved in multisensory attention and exhibits different connections to auditory, visual, somatosensory, and default mode networks, suggesting local specialization within these regions across multiple sensory modalities.^56^ The STS receives inputs from visual, auditory, and somatosensory areas and is involved in a series of behaviorally relevant processes, such as object recognition, speech, and face and voice integration.^57^^,^^58^

Sours et al.^48^ (2017) found that in newborns the IPS connects with visual and somatosensory areas, whereas the STS connects with visual, auditory, and somatosensory regions; all these connections appeared to be very similar to those of adults (see Figure 2, panel C). Thus, this evidence suggests that at the earliest stages of development, the brain already exhibits patterns of crossmodal connectivity that remain stable in adulthood.

What do the studies reviewed so far tell us about the origins of multisensory processing in humans?

First, it is important to revisit the difference in terminology presented in the introduction. The current literature investigated the development of multisensory abilities with different experimental paradigms and physiological techniques. The neurophysiological and electrophysiological techniques (EEG, fNIRS, and rs-fMRI) show the existence of a multisensory cortical and subcortical substrate present at or near birth, as documented by different brain responses (at a local or a circuit level) to unisensory and multisensory stimuli.^45^^,^^47^^,^^48^ On the one hand, the fact that multisensory areas are already present near birth and refine their functioning in the first months of life, parallels findings in the animal model.^1^^,^^48^^,^^59^ On the other hand, lack of behavioral data associated with neural data, do not allow stating that multisensory integration is present at birth. Similarly, behavioral paradigms (typically using looking times to assess preference) have revealed greater attention and sensitivity to multisensory events, as compared to modality-specific, unisensory, stimuli.^17^^,^^18^^,^^19^ However, such effects cannot be labeled as evidence of multisensory integration given the lack of a neural marker, such as the multisensory enhancement of brain activity in areas of sensory convergence. Thus, it seems that so far our knowledge of the development of multisensory integration (and specifically the integrative properties of multisensory neurons and its correlation with overt behavior) has been dominated by animal models, in which single cell recording is possible. Recognizing the difficulties of adopting the neurophysiological model of multisensory integration during human development, the question is it really so crucial to establish multisensory integration at birth in human beings? How would such knowledge benefit the newborn and infant human being whose overt behavior is very limited?

Our review has provided evidence that the functional architecture needed for multisensory processing in the mature brain is already present within the first days/weeks of life. We believe that this organization – in accord with animal and deprivation studies^3^^,^^9^^,^^11^^,^^12^^,^^60^^,^^61^ - may prepare the infant to receive, process and integrate various sensory inputs. As in adults, synchronous multisensory experience drives functional coupling between the occipital and temporal regions, in turn enhancing unisensory processing. Furthermore, multisensory information appears to increase activation in the two stimulated sensory cortices, which produce a higher signal with respect to activity when only one sensory region is stimulated.^47^^,^^51^^,^^48^

While this pattern suggests that the human brain is already ‘prepared’ for multisensory processing, in turn displaying a basic functional structure that might support an early sensitivity to multisensory information (see^59^ for a summary of the behavioral studies reported above), there is a large amount of evidence that innatism views of development should be abandoned in favor of more experience-dependent and gene-environment interactive views.

Sensory deprivation provides a case to demonstrate that it is only after considerable experience during early life with the statistics of multisensory events that the brain can skillfully use the basic principles of multisensory integration, such as space and time.^62^ For example, cats reared in the dark^9^ and human infants born with dense bilateral cataracts^63^ do not typically develop adult multisensory cortical circuits. This is because congenital visual deprivation influences the ability of cortical areas to process different sensory inputs, favoring reorganization of the visual cortex and, in turn, the functioning of the other sensory areas (both auditory and tactile areas, see, e.g.,^64^^,^^65^). Similar conclusions were obtained from deafferented individuals, born deaf and implanted with one or two cochlear implants at different stages of development. Stevenson et al.^66^ (2017) emphasized the role of sensitive periods of multisensory development and the need to implant as early as possible to allow children to optimize those crossmodal interactions supporting communication skills.

The complexity and outcome of multisensory processing and the behavioral responses derived from them are further influenced by genes. For instance, studies conducted across different neuropsychiatric disorders have revealed that disturbed expression levels of specific genes during critical periods of development may lead to hyperplasticity within the sensory circuits,^67^ leading to abnormal and hypersensitivity to sensory stimulation, such as autism spectrum disorder. Furthermore, it is worth noting that social functioning is multisensory in nature, as social cues need to be gathered by integrating facial expressions, speech, and body language. The integration of such cues occurs at a high level of multisensory integration to allow an appropriate behavioral response, and because social dysfunction is a common symptom across neuropsychiatric disorders, impaired multisensory processing might be one of the causes of social dysfunction.^68^

These findings underscore the importance of exposure to multisensory environments, which integrate with genes and allow for the optimal development of both sensory systems and their interactions. Therefore, having a brain endorsed with primitive multisensory mechanisms at birth is not sufficient, as its development is a long and multifactorial process.

In the following and last paragraph, we wish to provide some insight on how early sensitivity to multisensory experiences might prove helpful in situations of infants at risk of neurodevelopmental delays (or disorders), as is the case of preterm infants, who present a higher probability with respect to full-term infants to present delayed and atypical cognitive and socio-affective development, that might not even resolve with age.

Studies conducted in populations with diagnosed neurodevelopmental disorders, such as autism (ASD) and ADHD, have shown that one of the core characteristics of these conditions is the disruption of multisensory processing.^69^^,^^70^ This research has suggested that major difficulties in cognitive and social functioning correlate with the efficiency of multisensory integration.^71^^,^^72^^,^^73^ Assuming a cascade model, for example in ASD or ADHD, atypical processing of sensory information would impair multisensory integration, in turn affecting higher-order functions that depend on it. It follows that interventions aimed at refining multisensory integration could impact in a bottom-up manner on the development of cognitive and social abilities in neurodevelopmental disorders.^69^^,^^74^

In light of this evidence, in the last decades, multisensory-based rehabilitation programs have been promoted as a useful approach to neurological and neuropsychological rehabilitation.^75^^,^^76^^,^^77^ For example, infant massage (IM), by providing various sensory stimuli (e.g., touch, kinesthetic manipulation, voice, and facial expressions of the caregiver), can foster intensive and affective multisensory stimulation. The IM paradigm has been shown to have a positive and profound effect on visual system development (e.g., visual acuity and stereopsis maturation) in both preterm and Down syndrome children,^78^^,^^79^ as well as to facilitate the process of maturation of brain electrical activity in low-risk preterm infants similar to that observed (*in utero*) in term infants.^80^ It also has been shown that multisensory-integration-based interventions can significantly decrease autistic mannerisms^81^^,^^82^^,^^83^ and significantly improve the concentration level of children with ADHD and, in turn, improve the behavioral symptoms of impulsive-hyperactivity and hyperactivity.^74^

Although a causal link between multisensory integration impairments and the emergence of neurodevelopmental problems has not yet been established, evidence of a positive influence of multisensory training on cognitive and social functioning suggests a strong relationship between them.

## Clinical implications for studying the early development of multisensory processes: Insights from preterm infants

Recently, there has been growing interest in understanding the cognitive, motor, and socio-affective outcomes of preterm infants, who are at risk for brain injury and delayed development, which are sometimes associated with long-term deficits across different physical and cognitive domains.^84^^,^^85^ In particular, advances in neuroimaging techniques have allowed the observation of widespread alterations in cortical mass, surface area as well and whole brain connectivity^86^^,^^87^^,^^88^^,^^89^ in preterm infants, which in turn represent predictors of later deficits in cognitive processing and psychiatric diagnosis.

Interestingly, brain responses to multisensory stimuli differ significantly between preterm and full-term infants, and these differences are predictive of later behavioral deficits. In one study, Maitre et al.^90^ (2020) recorded continuous EEG in preterm and full-term infants during unisensory tactile, auditory, and audio-tactile (multisensory) stimulation. A questionnaire assessing internalizing and externalizing tendencies was administered to the families to observe the long-term consequences of prematurity at the age of 24 months. A series of differences between the groups emerged across ERP waveforms and topography: while multisensory processing in full-term infants was characterized by a linear addition of unisensory signals and by a single ERP topography for both summed unisensory and bimodal stimuli, preterm infants showed non-linear neural responses across multiple topographies. Furthermore, atypical brain activity predicted internalizing problems in preterm toddlers at 24 months of age, suggesting that dysfunctional multisensory processing in infancy may have long-term consequences on later behavior.

If preterm infants display atypical multisensory processing, which, in turn, appears to be crucial for typical development, early multisensory interventions might promote healthy cognitive and behavioral development. Some evidence in support of this hypothesis is offered by studies investigating the effects of multisensory exposures on a series of indexes of healthy neurodevelopment, such as motor, cognitive, and emotional behavior.

For example, the audio-tactile-visual-vestibular (ATVV) intervention consists of 10 min of auditory (a female voice) and tactile (massage) stimulation, followed by a 5-min vestibular stimulation (horizontal rocking), which has proved useful in improving alertness and feeding behavior (see^91^ for a recent review; ^92^^,^^93^).

Another intervention, the Family Nurture Intervention (FNI) was designed to facilitate affective connections between infants and their mothers by adopting a multisensory approach to promote such bonding.^94^ In particular, the FNI consists of a 1-h interaction with the infant, during which the caregiver is required to establish eye contact (visual stimulation), perform vocal soothing (auditory stimulation), and skin-to-skin contact (tactile stimulation). Welch et al.^95^ (2014) found that preterm infants treated with such multisensory intervention showed increased EEG power in the frontal polar region across several frequencies, as assessed during sleep, and that such cerebral pattern is commonly associated with healthy neurodevelopment.

In a follow-up study, the same authors^96^ found that at 18 months from preterm birth, FNI improved sensorimotor development, concept formation, memory, and language as assessed through the Bayley Scale III, a standardized and validated assessment of general infant development. The FNI also improved behavioral problems, as assessed on the Child Behavior Checklist, which measures externalizing and internalizing behavior, as well as emotional, attention, sleep, anxiety and aggression problems in very early childhood.

The above-mentioned studies present a series of limitations that preclude these interventions from being considered conclusive. Indeed, as observed in a recent meta-analysis,^97^ the sample sizes used in most studies are very small, and the longitudinal data are poor, leaving the question of whether these interventions are beneficial in the long term.

Considering the increasing number of preterm births, there is the urge to intervene immediately to promote the healthy development of these infants in a period in life in which brain plasticity can support faster and more efficient learning.

Furthermore, multisensory interventions developed so far capitalize on two decades of studies showing how multisensory learning can benefit individuals as our brains have evolved to process the natural (multisensory) environment.^98^ As shown in the first paragraph of this chapter, human infants are naturally attracted to multisensory stimuli, and their brains appear to be tuned to process such information. This is reflected in their facilitation in processing multisensory information over unisensory information, which is a pattern that persists into adulthood. As shown by behavioral, neuroimaging and computational data,^99^^,^^100^^,^^101^ multisensory learning outperforms unisensory learning, and alters the underlying multisensory structures, as well as the connectivity between unisensory structures. Thus, promoting multisensory training in preterm infants holds promise for the development of healthy and successful individuals.

**Figure 3:**
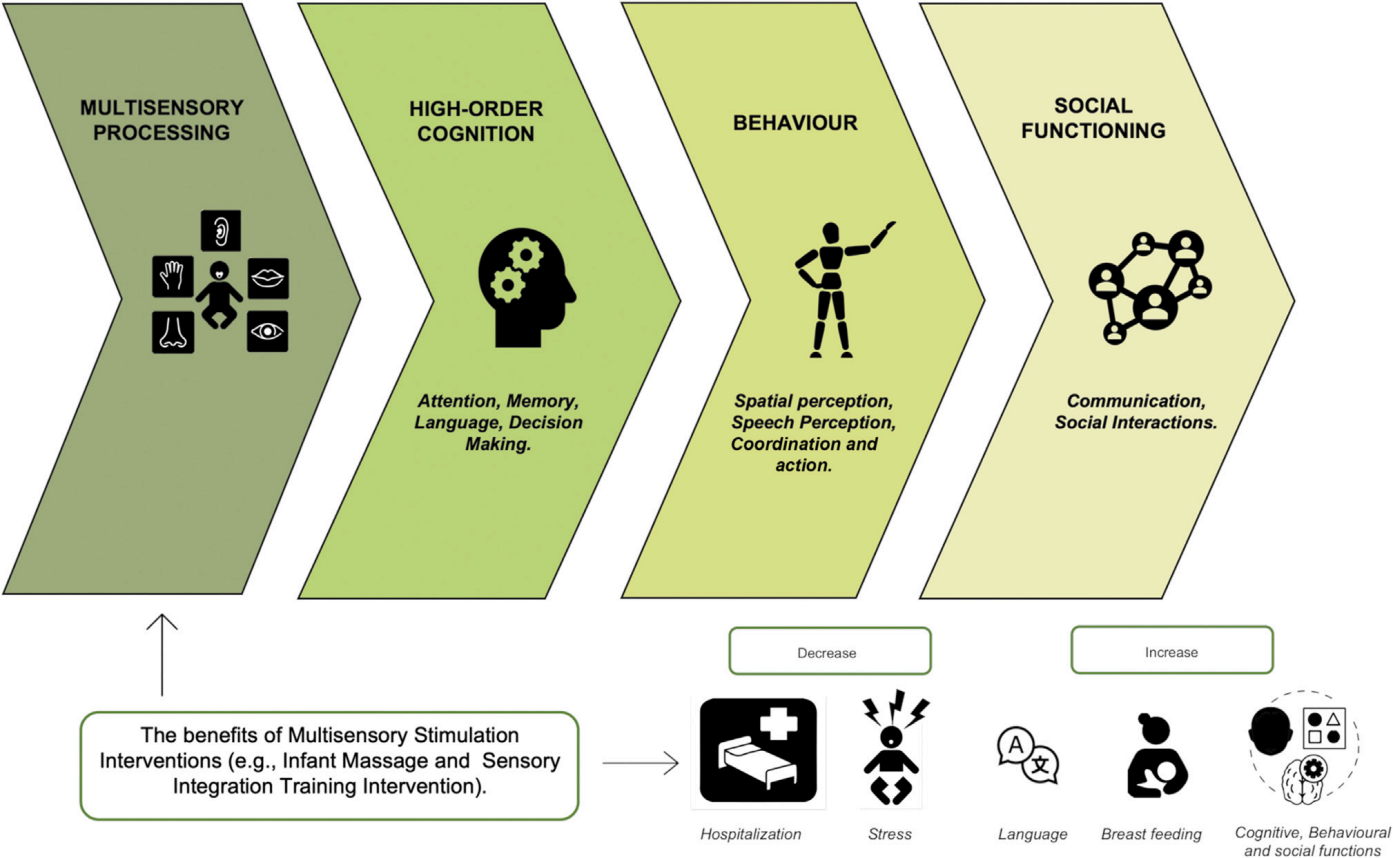
The graph depicts a suggestion on how multisensory processing could influence higher-order cognition, in turn impacting how individuals will benefit from it on a behavioral and functional level

In conclusion, this review has illustrated that since birth, human newborns step into the world with a brain that is already prepared to receive and process multisensory stimuli. Throughout postnatal life, multisensory experience shapes and reinforces neural circuits that are laid down for processing multisensory stimuli. We have concluded the review with insights from the preterm literature and demonstrated the potential benefits of multisensory stimulation in the clinical setting. Indeed, we believe that this is the path multisensory research should take to address the question of promoting healthy development in newborns and infants who are born at risk of developmental delays (Figure 3).
